# Supplementary material for: Snap29 Is Dispensable for Self-Renewal Maintenance but Required for Proper Differentiation of Mouse Embryonic Stem Cells
Source: Int J Mol Sci. 2023 Jan 1;24(1):750. doi: 10.3390/ijms24010750 (PMC9821219; doi:10.3390/ijms24010750)
Supplement: Supplementary file 1 [file ijms-24-00750-s001.zip › ijms-1963756-supplementary.pdf]

Supporting Information

Snap29 Is Dispensable for Self-Renewal Maintenance but Required for Proper Differentiation of Mouse Embryonic Stem Cells

Yumei Jia [1], Zhaoyuan Guo [1], Jiahao Zhu [1], Guanyu Qin [1], Wenwen Sun [1], Yu Yin [2], Haiying Wang [3] and Renpeng Guo [1,*]

[1] College of Food Science and Technology, Nanjing Agricultural University, Nanjing 210095, China
[2] Yunnan Key Laboratory of Primate Biomedical Research, Institute of Primate Translational Medicine, Kunming University of Science and Technology, Kunming 650500, China
[3] State Key Laboratory of Medicinal Chemical Biology, Department of Cell Biology and Genetics, College of Life Sciences, Nankai University, Tianjin 300071, China
* Correspondence: guorp@njau.edu.cn

The Following Files are Included:

Supplementary Figures and Legends

Supplementary Figures S1–S3

Supplementary Tables S1–S3

Supplementary Figures and Legends

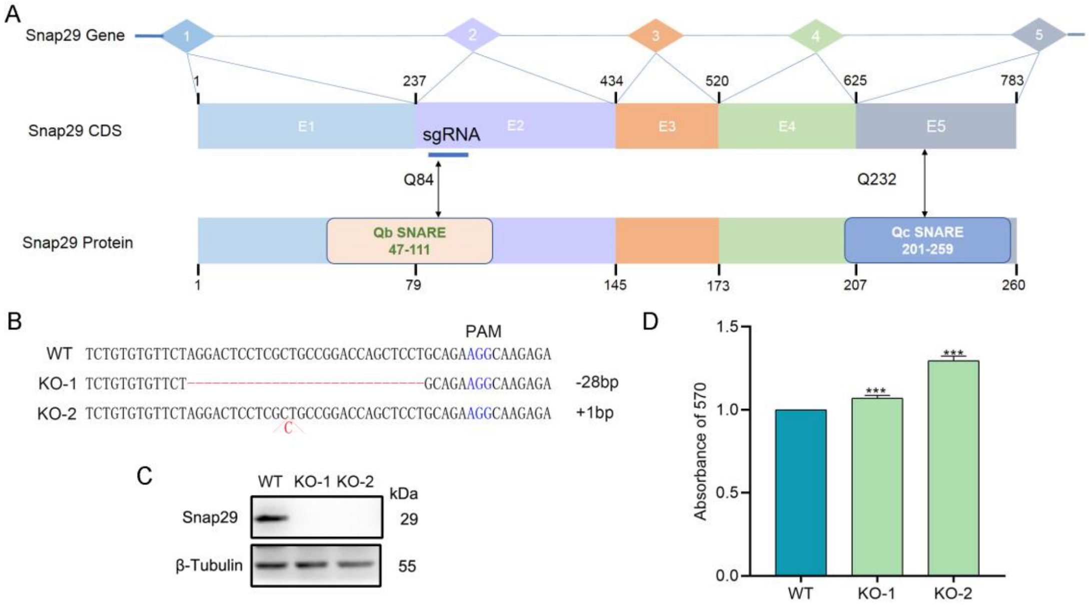

Supplementary Figure S1. Targeted disruption of *Snap29* with CRISPR/Cas9 in mouse ESCs.

(A) Illustration of mouse *Snap29* gene, its coding sequence (CDS) and Snap29 protein. The sgRNA was designed for targeting the second exon of *Snap29*.

(B) Sequencing data of *Snap29* mutations induced by CRISPR/Cas9 in ESC clones. Deletions are indicated by dashes, and PAM is shown in blue.

(C) Western blot analysis of Snap29 protein levels in *Snap29*$^{+/+}$ (WT) and *Snap29*$^{-/-}$ (KO-1 and KO-2) ESCs.

(D) MTT assay of WT, KO-1 and KO-2 ESCs. Mean ± SEM from three independent experiments. ***$P < 0.001$, unpaired two-tailed Student's t-test, compared with the WT group.

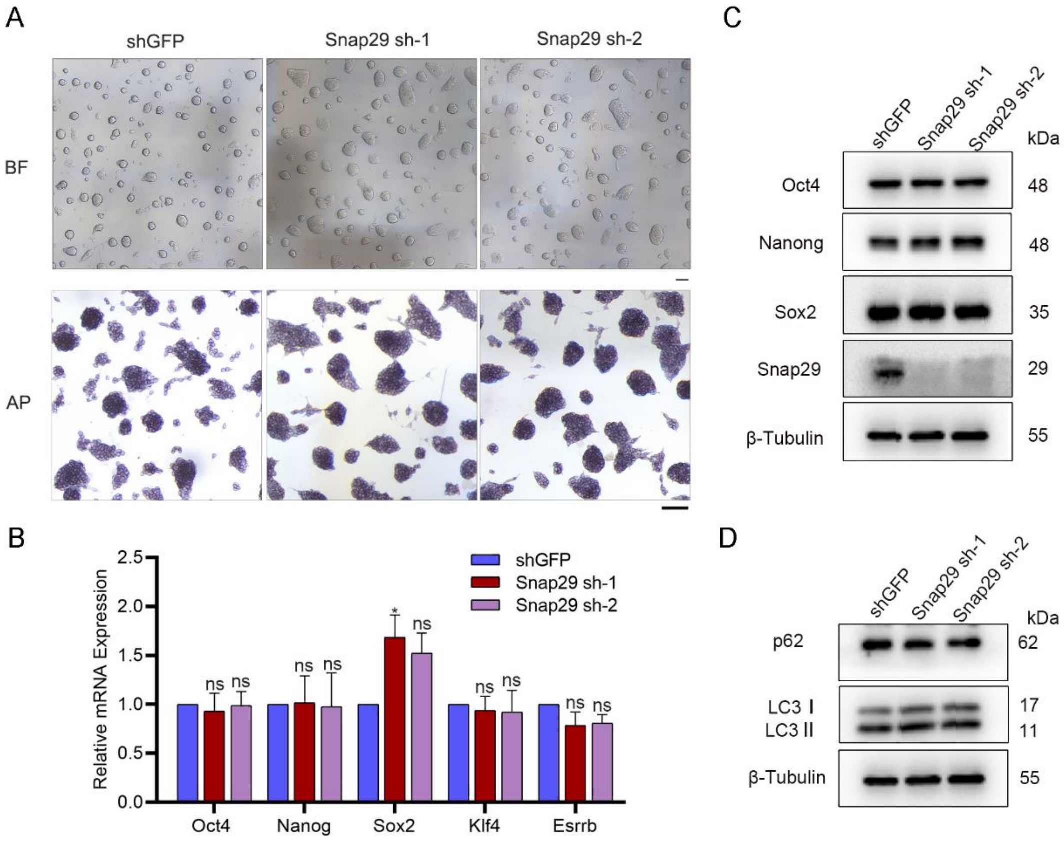

Supplementary Figure S2. *Snap29* silencing does not damage self-renewal or autophagy of ESCs.

(A) Morphology of control (shGFP) and *Snap29* knockdown ESCs under bright field (BF) and alkaline phosphatase (AP) staining analysis. Scale bar, 100 μm.

(B) Relative mRNA levels of pluripotent marker genes measured by qPCR.

(C) Western blot analysis of Oct4, Nanog, Sox2 and Snap29 in control and *Snap29* knockdown ESCs.

(D) Western blot analysis of LC3 I/II and p62 in control and *Snap29* knockdown ESCs.

Mean ± SEM from three independent experiments. *$P < 0.05$, ns not significant ($P > 0.05$), unpaired two-tailed Student's *t*-test, compared with control groups.

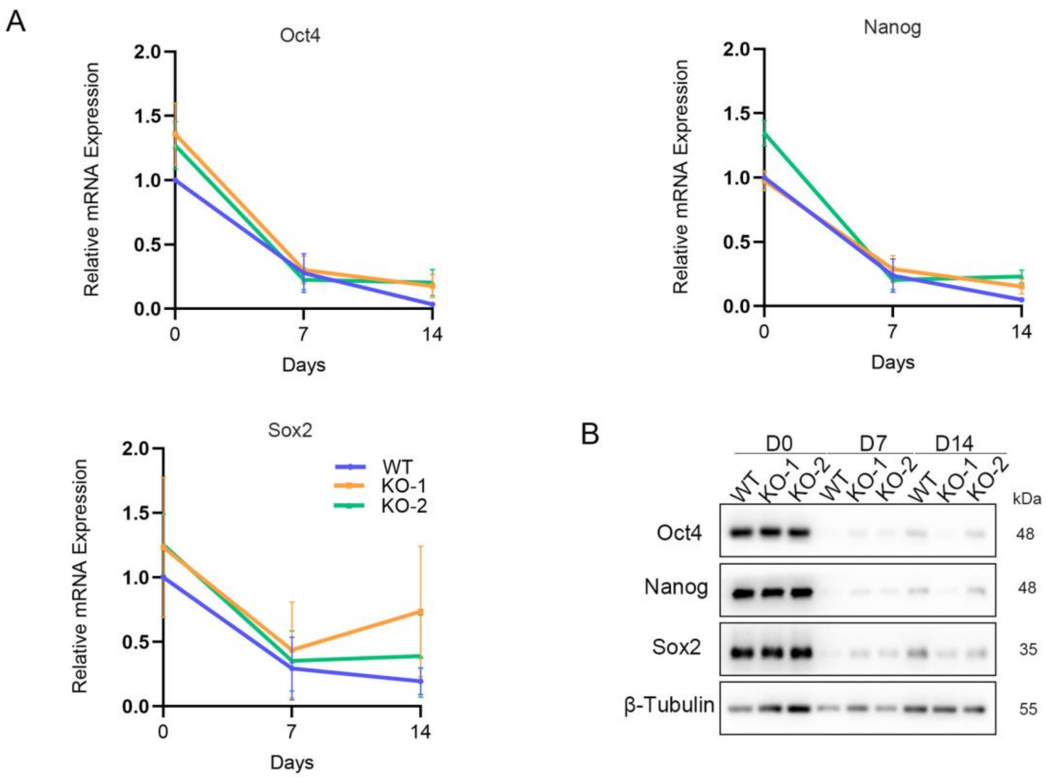

Supplementary Figure S3. *Snap29* depletion does not impact the down-regulation of pluripotent genes.

(A) Relative mRNA levels of pluripotent marker genes Oct4, Nanog and Sox2 at indicated time points of differentiation.

(B) Western blot analysis of Oct4, Nanog and Sox2 at indicated time points of differentiation.

Data represent mean ± SEM from three independent experiments.

Supplementary Table S1 Primers for qPCR analysis

Genes	Forward	Reverse
Oct4	TTGGGCTAGAGAAGGATGTGGTT	GGAAAAGGGACTGAGTAGAGTGTGG
Sox2	GCACATGAACGGCTGGAGCAACG	TGCTGCGAGTAGGACATGCTGTAGG
Nanog	TTGCTTACAAGGGTCTGCTACT	ACTGGTAGAAGAATCAGGGCT
Klf4	CCATCGGACCTACTTATCTGC	AAAACCTCAAACCAAAACCC
Esrrb	CAGGCAAGGATGACAGACG	GAGACAGCACGAAGGACTGC
Gata4	GCTATGCATCTCCTGTCACTCAGA	CCAAGTCCGAGCAGGAATTTGAAG
Gata6	CTTCTCCTTCTACACAAGCGACCA	ATACTTGAGGTCACTGTTCTCGGG
T	CATCGGAACAGCTCTCCAACCTAT	TACCATTGCTCACAGACCAGAGAC
Flk1	CCTGGTCAAACAGCTCATCA	AAGCGTCTGCCTCAATCACT
Hand1	AAGGATGCACAAGCAGGTGAC	TTTAATCCTCTTCTCGCCGGG
Fgf5	GAAACTCGGATACAGCATCCCTCT	GGATCGCTACAGAGAATCCCACTT
Nestin	CTGGATCTGGAAGTCAACAGAGGT	ATCCTCAGTTTCCACTCCTGTAGC
Cxcl10	CCAAGTGCTGCCGTCATTTTC	GGCTCGCAGGGATGATTTCAA
Actal	CCCAAAGCTAACCGGGAGAAG	CCAGAATCCAACACGATGCC
Trim54	GGAGAAGCAGCTCATTTGCC	CCTCCTGAAGACACCGTTGTG
Jam2	GTGCCCACTTCTGTTATGACTG	TTCCCTAGCAAACTTGTGCCA
Col5a2	TTGGAAACCTTCTCCATGTCAGA	TCCCCAGTGGGTGTTATAGGA
Fstl3	CTGCCTCCCCTGCAAAGATTC	CGGTACATGACGCGCAAGT
Snap29	AGGTTTACCCAAAGAACTCGACC	TGGCCTAATCCCACGGACA
Ttyh3	AGAAAGACAGACGAGCACCTT	CCGTAGAATCCCACTGCAATG
Il3Ra	CTGGCATCCCACTCTTCAGAT	GGTCCCAGCTCAGTGTGTA
Tubb4A	CACCTGCAAGCCGGTCAAT	TCCCCATGATAGGTCCCAGTG
Kif21B	CACGACAGCCCCATCAATG	CCGCCGTATGCTCCAGAAC
Cdx2	CAAGGACGTGAGCATGTATCC	GTAACCACCGTAGTCCGGGTA
Cacna1H	CGTGACACTGGGCATGTTC	CCACCATCTTGATAACCATCTCC
Ccnd2	GAGTGGGAACTGGTAGTGTTG	CGCACAGAGCGATGAAGGT
Hesx1	TGAGAGCATTTTAGGACTGGACC	GGGTGGGTTGCCATCTTTCT
Lonrf3	CGAGGTATGGGATGGCTTTAAG	GCTCCAGGCACAGTTTACAAA
Snap23	CGGGCTCACCAGGTTACTG	GGCTAAACCCAGGATTCTCCTT
Snap47	TCTGATATGCGTGTCCACTCC	GTGATCGCACCGAAGATAAAGAG
Gapdh	TCAACAGCAACTCCCACTCTTCCA	ACCACCCTGTTGCTGTAGCCGTAT

Supplementary Table S2 Antibodies

Antibody	Source	Cat. No.	Dilution (IF)	Dilution (WB)
Nanog	Bethyl	A300-397A	1:400	1:5000
Oct3/4	Santa Cruz Biotechnology	sc-5279	1:400	1:5000

SSEA1	Millipore	MAB4301	1:400	
Sox2	Emd millipore corporation	AB5603	1:400	1:5000
LC3B	HuaBio	ET1701-65		1:20000
p62	abcam	ab109012		1:4000
Snap29	abcam	ab138500		1:2000
β-Tubulin	HuaBio	EM0103		1:2000
AFP	HuaBio	R1601	1:100	1:1000
α-SMA	abcam	ab5694	1:200	1:2000
βIII-Tubulin	abcam	ab18027	1:200	1:2000

IF: immunofluorescence; WB: Western blot

Supplementary Table S3 Oligonucleotides used for knockout or knockdown

Oligonucleotides	Sequence	experiment
Snap29 sgRNA F	GTTCTAGGACTCCTCGCTGC	knockout
Snap29 sgRNA R	GCAGCGAGGAGTCCTAGAAC	
control shRNA F	gatccGGCGTTCAATTAGCAGACCATTCAAGAGATGGTCTGCTAATTGAACGCCTTTTTTAAGCTTg	shRNA knockdown
control shRNA R	aattcAAGCTTAAAAAAGCGTTCAATTAGCAGACCATCTCTTGAATGGTCTGCTAATTGAACGCCg	
Snap29 shRNA-1 F	gatccGATGAGCTGTCCGTGGGATTATTCAAGAGATAATCCCACGGACAGCTCATCTTTTTTg-	
Snap29 shRNA-1 R	aattcAAAAAAGATGAGCTGTCCGTGGGATTATCTCTTGAATAATCCCACGGACAGCTCATCg	
Snap29 shRNA-2 F	gatccGCAGATTGAAAGAAGCCATAAATTCAAGAGATTTATGGCTTCTTTCAATCTGTTTTTTg	
Snap29 shRNA-2 R	aattcAAAAAACAGATTGAAAGAAGCCATAAATCTCTTGAATTTATGGCTTCTTTCAATCTGCg	
siNC	UUCUCCGAACGUGUCACGUTT	siRNA knockdown
Snap23 siRNA-1	GGAAAGAACUAUAAGGCAATT	
Snap23 siRNA-2	GGAUAUGGGCAAUGAAAUUTT	
Snap23 siRNA-3	GCAAAGAAACUCAUUGAUATT	

Snap47 siRNA-1	UGAUAGGACUGAUGGCAAATT
Snap47 siRNA-2	GGAAGAUGUUGAUGAUAUTT
Snap47 siRNA-3	CUUGAGAAGCAAUGGGUUUTT
